# Unintentional Discontinuation of Chronic Medications for Seniors in Nursing Homes: Evaluation of a National Medication Reconciliation Accreditation Requirement Using a Population-Based Cohort Study

**DOI:** 10.1097/MD.0000000000000899

**Published:** 2015-06-26

**Authors:** Nathan M. Stall, Hadas D. Fischer, C. Fangyun Wu, Arlene S. Bierman, Stacey Brener, Susan Bronskill, Edward Etchells, Olavo Fernandes, Davina Lau, Muhammad M. Mamdani, Paula Rochon, David R. Urbach, Chaim M. Bell

**Affiliations:** From the Department of Medicine (NMS, EE, CMB), University of Toronto; Institute for Clinical and Evaluative Sciences (HDF, ASB, S Bronskill, MMM, PR, DRU, CFW, CMB); Keenan Research Centre (ASB, MMM), Li Ka Shing Knowledge Institute, St Michael's Hospital; Institute of Health Policy, Management and Evaluation (ASB, S Bronskill, EE, PR, DRU, CMB); Lawrence S. Bloomberg Faculty of Nursing (ASB), University of Toronto; Health Quality Ontario (S Brener); Department of Pharmacy (OF), University Health Network; Leslie Dan Faculty of Pharmacy (OF, MMM), University of Toronto; Division of General Internal Medicine (DL, CMB), Mount Sinai Hospital; Women's College Research Institute (PR), Women's College Hospital; and Department of Surgery (DRU), University of Toronto, Toronto, Canada.

## Abstract

Transitions of care leave patients vulnerable to the unintentional discontinuation of medications with proven efficacy for treating chronic diseases. Older adults residing in nursing homes may be especially susceptible to this preventable adverse event. The effect of large-scale policy changes on improving this practice is unknown.

The objective of this study was to analyze the effect of a national medication reconciliation accreditation requirement for nursing homes on rates of unintentional medication discontinuation after hospital discharge.

It was a population-based retrospective cohort study that used linked administrative records between 2003 and 2012 of all hospitalizations in Ontario, Canada. We identified nursing home residents aged ≥66 years who had continuous use of ≥1 of the 3 selected medications for chronic disease: levothyroxine, HMG-CoA reductase inhibitors (statins), and proton pump inhibitors (PPIs).

In 2008 medication reconciliation became a required practice for accreditation of Canadian nursing homes.

The main outcome measures included the proportion of patients who restarted the medication of interest after hospital discharge at 7 days. We also performed a time series analysis to examine the impact of the accreditation requirement on rates of unintentional medication discontinuation.

The study included 113,088 adults aged ≥66 years who were nursing home residents, had an acute hospitalization, and were discharged alive to the same nursing home. Overall rates of discontinuation at 7-days after hospital discharge were highest in 2003–2004 for all nursing homes: 23.9% for thyroxine, 26.4% for statins, and 23.9% for PPIs. In most of the cases, these overall rates decreased annually and were lowest in 2011–2012: 4.0% for thyroxine, 10.6% for statins, and 8.3% for PPIs. The time series analysis found that nursing home accreditation did not significantly lower medication discontinuation rates for any of the 3 drug groups.

From 2003 to 2012, there were marked improvements in rates of unintentional medication discontinuation among hospitalized older adults who were admitted from and discharged to nursing homes. This change was not directly associated with the new medication reconciliation accreditation requirement, but the overall improvements observed may have been reflective of multiple processes and not 1 individual intervention.

## Abstract 2

Supplemental Digital Content is available in the text

## BACKGROUND

Transitions of care, such as admission to and discharge from hospital, leave patients vulnerable to preventable adverse events due to poor communication.^1^ One such event is prescription medication errors of omission, including the unintentional discontinuation of medications when transitioning between settings. For example, a prescription renewal is overlooked in a patient who had been regularly receiving a medication with proven efficacy for treating chronic disease.^2,3^ Indeed, over two thirds of patients admitted to hospitals have unintended medication discrepancies,^4^ and these discrepancies remain common at discharge.^5,6^ A systematic review of these medication errors reported that over half have the potential for harm,^4^ and a prospective cohort study revealed that >1 in 10 patients experience an adverse drug event (ADE) following hospital discharge.^7^ Importantly, more than half of all hospital medication errors occur at the interfaces of care.^8^ This issue is of critical importance, with ADEs accounting for significant increases in health services utilization and costs,^9^ and approximately 7000 deaths annually in the United States alone.^10^

Much of the research on transition of care-related ADEs has centered on the transition between acute care hospitals and the community; few studies have considered the transition between acute care hospitals and nursing homes.^11,12^ This is of concern because older adults residing in nursing homes may be especially vulnerable to transition of care-related medication discontinuation.^13^ As a result of their frail and comorbid state, nursing home residents commonly experience deteriorations in health status necessitating frequent transfers to and from acute care facilities.^14,15^ Moreover, these individuals suffer from multiple chronic conditions, which are commonly managed long-term with prescription medications. Adherence to clinically appropriate evidence-informed therapies is important for lowering the risk of progression and complications related to their underlying chronic conditions. This concept must be balanced with concerns about polypharmacy and medication overuse.

Recognizing this patient safety issue, medication reconciliation—the formal process for identifying and correcting unintended medication discrepancies across transitions of care—has emerged and has been widely endorsed.^16,17^ The practice is now mandated by health care accreditation bodies in both the United States and Canada across the continuum of care.^18,19^ In Canada, nursing homes were among the last health care institutions to be evaluated on this intervention, having become a required practice for accreditation in 2008. This provided a unique opportunity to assess the effect of new accreditation requirements on rates of discontinuation of medications for chronic diseases in seniors admitted from and discharged to nursing homes.

## METHODS

### Study Overview

We conducted a population-based retrospective cohort study between May 1, 2003, and February 28, 2012, of all hospitalizations from nursing homes in Ontario, Canada, to identify residents aged ≥66 years who had continuous use of ≥1 of 3 selected medications for chronic disease: levothyroxine, HMG-CoA reductase inhibitors (statins), and proton pump inhibitors (PPIs).

The primary outcome of interest was the failure to refill medication prescriptions within 7 days after discharge from hospital and return to the same nursing home. This outcome is a reliable and objective measure of adherence in large patient groups.^20^ We analyzed this outcome both before and after the 2008 inclusion of a medication reconciliation program for accreditation of nursing homes.^21,22^ We performed a time series analysis to examine the impact of nursing home accreditation on medication discontinuation rates.

### Data Sources

The study was conducted in Ontario, Canada, using linked, deidentified population-based administrative databases held at the Institute for Clinical Evaluative Sciences (ICES). These databases have been used extensively in prior research of medication use among older individuals in nursing homes.^23,24^ The data sets are linked via encrypted health care numbers and include: the Ontario Drug Benefit program database, which contains detailed drug information for Ontario's >1.5 million older adults (and all residents of nursing homes); Canadian Institute for Health Information Discharge Abstract Database, which documents all hospitalizations and procedures in Ontario hospitals; the Registered Persons Database, which contains demographic data for all of Ontario's residents who have ever had a valid health card number in Ontario's universal single-payer health care system; the Ontario Health Insurance Plan database, which includes physician billing claims for visits and procedures performed within Ontario. This study was approved by the research ethics board of the Sunnybrook Health Sciences Centre and included a waiver of patient consent.

### Derivation of Patient Group and Medication Cohorts

**FIGURE 1:**
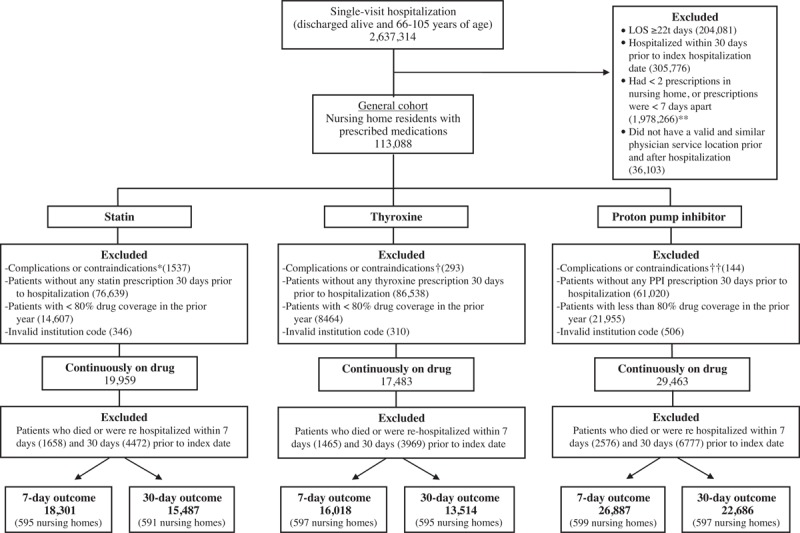
Derivation of patient and medication groups. LOS = length of stay.

The population consisted of nursing home residents aged ≥66 years who were hospitalized and discharged alive from an acute care hospital between May 1, 2003, and February 28, 2012 (Figure 1).

Patients were excluded if their hospital length of stay (LOS) was >21 days or if they had a hospitalization within 30 days before their index hospitalization admission date, to best isolate the effect of the index hospitalization on our outcomes of interest. In order to select patients who had been in a nursing home for ≥7 days, we also excluded patients if they had <2 prescriptions in a nursing home or if the prescriptions were <7 days apart. Finally, we excluded patients who were not admitted from and discharged to the same nursing home to avoid differences in institutional practices.

Medication continuity for patients who had been dispensed levothyroxine, HMG-CoA reductase inhibitors (statins), or PPIs for ≥1 year prior to hospital admission was examined. All of these medications are commonly used in older adults, have documented adherence estimates for large populations,^25–27^ and are evidence-based therapies with established long-term efficacy.^28–30^ They also include medications for the treatment of symptomatic and asymptomatic diseases. Additionally, a structured modified Delphi panel process identified adherence to these 3 medications as consensus quality indicators to evaluate medication continuity between hospitals and nursing homes.^31^ Earlier work using both a structured chart review^2^ and a population-based cohort study using the same administrative records utilized in the present study^3^ reported significant unintentional discontinuation of the above 3 medication groups at hospital discharge.

A continuous medication user was defined by ≥1 year of filled prescriptions without interruption beyond an allotted grace period of 20%.^31^ We used the Ontario Drug Benefit database to estimate the intended duration of each prescription, and patients were required to demonstrate 80% coverage for a minimum of 1 year of use of the medication (total days of supply in the year before admission of ≥292 days) for study entry. Patients were also excluded if they had a medication-specific complication (eg, rhabdomyolysis for statins) or an ADE (Appendix 1 for full details, http://links.lww.com/MD/A281).

### Exposures: Accreditation of Nursing Homes

In 2008, Accreditation Canada made medication reconciliation a required practice for accreditation of nursing homes.^22^ The 2 required practices were medication reconciliation at admission and at transfer or discharge. These processes included completing a Best Possible Medication History and communicating up-to-date medication lists to the next care provider; they complemented analogous efforts in acute care hospitals that were previously implemented.^22^ National compliance rates with the medication reconciliation required practices in nursing homes were 67%, 64%, and 69% in 2009, 2010, and 2011, respectively.^22^ This initiative was also supported by Safer Healthcare Now!,^32^ a program of the Canadian Patient Safety Institute.^21,33^

Our exposure of interest was discharge from hospital to a nursing home that was successfully accredited by Accreditation Canada following institution of the new policy, allowing us to assess the impact of the medication reconciliation requirement.^21^ We obtained the dates of accreditation directly from Accreditation Canada. In our cohort, February 11, 2008, was the date the first nursing home was accredited after medication reconciliation became a required practice, and November 19, 2010, was the date the last nursing home was accredited. These dates comprised the initial policy implementation period.

This methodology allowed us to categorize hospitalizations from nursing homes into the following groups: discharges to a nursing home that was not accredited during our implementation period, discharges to an accredited nursing home before the known date of accreditation, discharges to an accredited nursing home after the known date of accreditation, and discharges to an accredited nursing home with an unknown date of accreditation (some nursing homes did not provide consent to release their dates of accreditation). For this latter group, patients who had a hospital discharge date before the date the first nursing home was accredited (February 11, 2008) were aggregated into the second group (individuals who were discharged from hospital before the known date of accreditation of their facility). Patients who had a hospital discharge date after the date the last nursing home was accredited (November 19, 2010) were aggregated into the third group (individuals who were discharged from hospital after the known date of accreditation of their facility). Finally, patients who had a hospital discharge date between the start and finish dates of accreditation (February 11, 2008, to November 19, 2010) were removed from the analysis. We were therefore left with 3 categories of interest: discharges to a nursing home that was not accredited during our study period, discharges to an accredited nursing home before the known date of accreditation, and discharges to an accredited nursing home after the known date of accreditation.

### Outcomes: Assessment of Unintentional Medication Discontinuation

Our primary outcome of interest was the proportion of patients who were dispensed the medication of interest within 7 days after hospital discharge. This 7-day time period was selected because in Ontario, Canada, the drug benefit program allows pharmacies to provide nursing home residents with 7 days’ supply of medications. This time frame therefore accounted for any leftover medication before the hospital admission. Our secondary outcome of interest was the proportion of patients who were dispensed the medication of interest within 30 days after hospital discharge, allowing health care professionals more time to reconcile medications after a patient's return to a nursing home.

For these analyses, depending on the outcome of interest, we derived 2 subcohorts from each of the 3 medication group cohorts: a cohort that excluded patients who died or were rehospitalized within 7 days, and a cohort that excluded patients who died or were rehospitalized within 30 days (Figure 1). These exclusions allowed us to ensure that when individuals returned to their nursing home, they had ample opportunity for their medications to be restarted.

### Statistical Analysis

The proportion of patients who restarted the medication of interest after hospital discharge was calculated for each fiscal year from May 1, 2003, to February 28, 2012. We initially used χ^2^ tests to compare the rates of unintentional medication discontinuation with a discharge date before and after accreditation of the nursing home (Table 1). A *P* value of *P* < 0.05 was considered statistically significant.

**TABLE 1:**
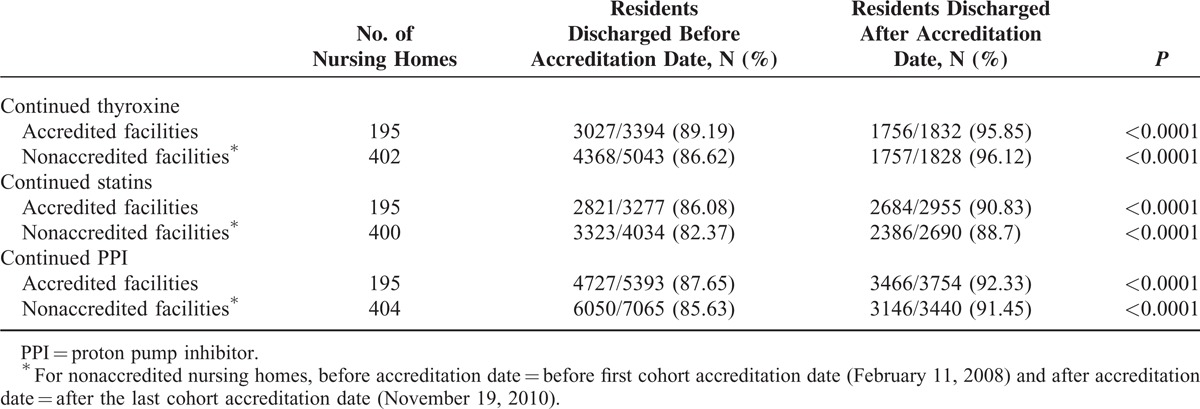
Medication Continuation Before and After the Medication Reconciliation Program and Quality Improvement Strategy (Fiscal Years 2003–2011)

We subsequently conducted a cross-sectional time series analysis to examine the impact of nursing home accreditation on medication discontinuation within 7 days of discharge during the study time frame of May 1, 2003, to February 28, 2012.^34–36^ Because each nursing home had a different accreditation date and ranged from February 11, 2008, through November 19, 2010, we labeled the accreditation date of each of the accredited nursing homes as time “0.” For the nonaccredited nursing homes, we randomly assigned an accreditation date that fell between the start and end of the accreditation period based on the distribution of accreditation dates of the accredited nursing homes during this period.^37^ Using time “0” as the reference, we looked back 20 quarters from this timepoint for each nursing home to establish historical trends and 6 quarters following time “0” to assess changes in the historical trends that may be attributable to the accreditation. In each quarter, we examined the continuation rate of chronic medications following hospitalizations occurring during the given quarter being studied. We used interventional autoregressive integrated moving average (ARIMA) models to assess the impact of accreditation (time “0”) on the rate of discontinuation of chronic drugs. This type of analysis is a robust method of accounting for existing trends in the data.^36,38^ Model specification was guided by inspection of correlograms. Autocorrelation was assessed using the Ljung–Box χ^2^ statistic, and stationarity was assessed using the augmented Dickey–Fuller test.^39,40^ The ARIMA models were developed for the accredited and nonaccredited data separately. All the analyses were performed independently in each of the 3 drug exposure groups. All statistical analyses were conducted in SAS software version 9.3 (SAS Institute Inc, Cary, NC).

## RESULTS

The study included 113,088 nursing home residents aged ≥66 years from 722 unique facilities. They were all discharged alive from an acute care hospitalization and returned to the same nursing home between May 1, 2003, and February 28, 2012 (Figure 1). The mean age was 84 years, and about two thirds were women (Table 2). Patients were prescribed a mean of nearly 16 different drugs in the year prior to their index hospitalization. The average LOS for the cohort's index hospitalization was 6.25 days, and 5.3% of these encounters involved an intensive care unit admission.

**TABLE 2:**
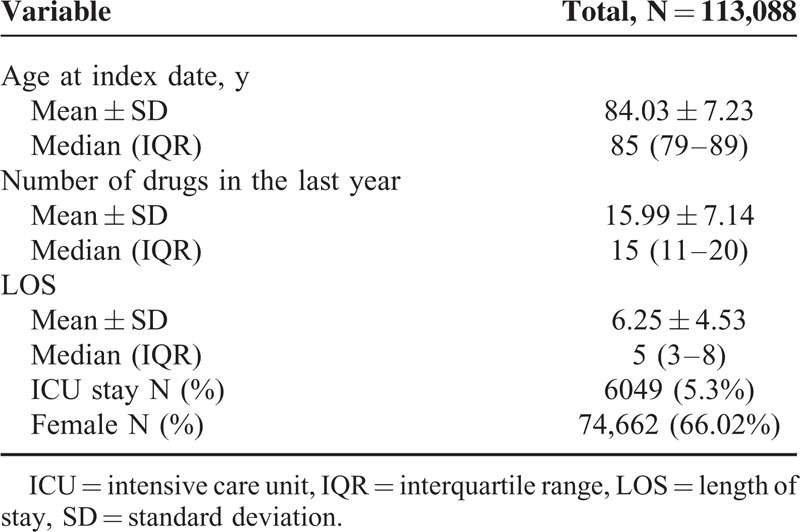
Patient Characteristics

### Rates of Medication Discontinuation Over Time

**FIGURE 2:**
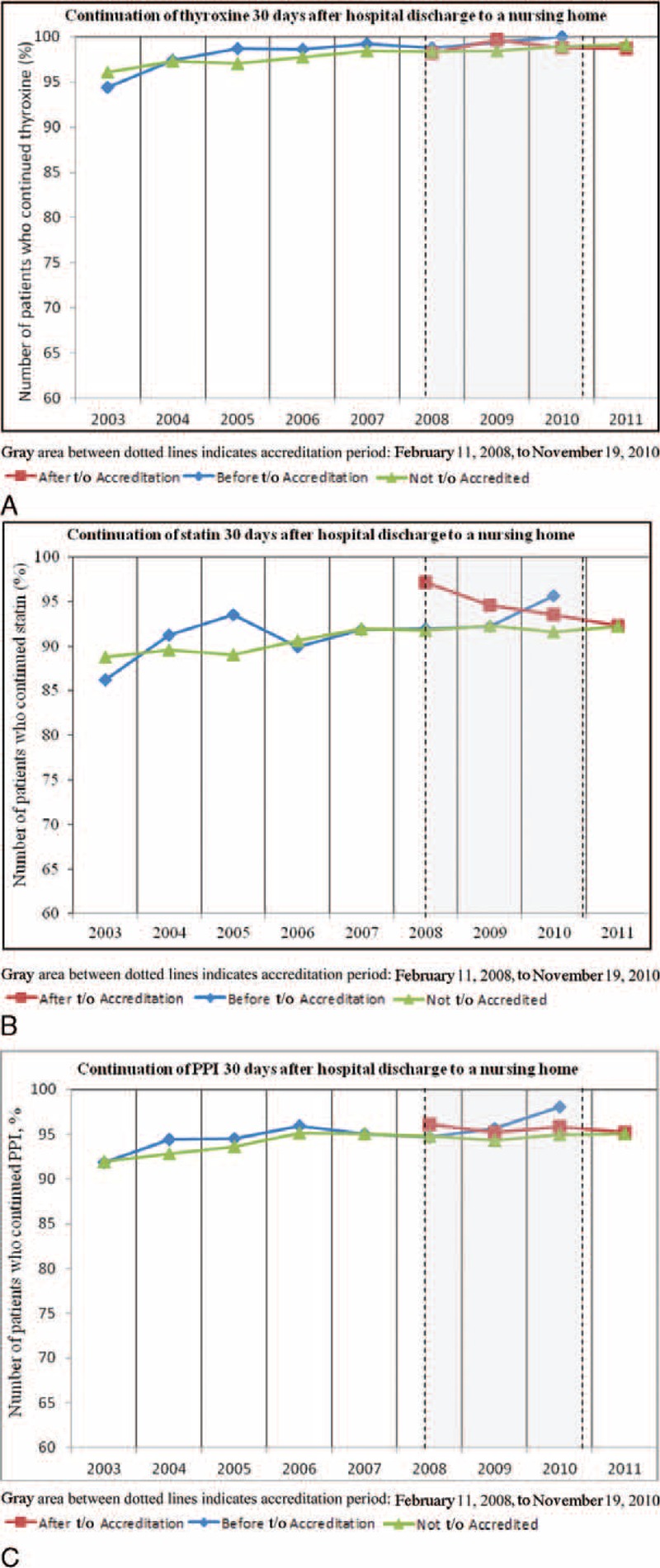
(A) Continuation of thyroxine 7 days after hospital discharge to a nursing home (fiscal years 2003–2011). Gray area between dotted lines indicates accreditation period: February 11, 2008, to November 19, 2010. (B) Continuation of statin 7 days after hospital discharge to a nursing home (fiscal years 2003–2011). Gray area between dotted lines indicates accreditation period: February 11, 2008, to November 19, 2010. (C) Continuation of PPIs 7 days after hospital discharge to a nursing home (fiscal years 2003–2011). Gray area between dotted lines indicates accreditation period: February 11, 2008, to November 19, 2010. PPI = proton pump inhibitor.

Rates of unintentional medication discontinuation in older adults admitted from and discharged to all nursing homes improved for all 3 medications between 2003–2004 and 2011–2012 (Figure 2A–C). At 7 days after hospital discharge (our primary outcome), overall rates of discontinuation were the highest in 2003–2004. They were 23.9% for thyroxine, 26.4% for statins, and 23.9% for PPIs. In most cases, these rates decreased annually and were the lowest in 2011–2012. They were 4.0% for thyroxine, 10.6% for statins, and 8.3% for PPIs. Rates of discontinuation at 30 days after hospital discharge (our secondary outcome) were lower overall but followed a similar trend over the 9-year study period (Appendix 2A–C, http://links.lww.com/MD/A281*)*.

### Rates of Medication Discontinuation Before and After Accreditation Requirement

Rates of medication discontinuation were compared before and after the date of accreditation. For all 3 medications, rates of discontinuation at 7 days after hospital discharge were significantly lower after accreditation (Table 1). However, rates were also significantly lower for nonaccredited nursing homes before and after the dates spanning accreditation (February 11, 2008, to November 19, 2010).

### Time Series Analysis of Accreditation Requirement

**FIGURE 3:**
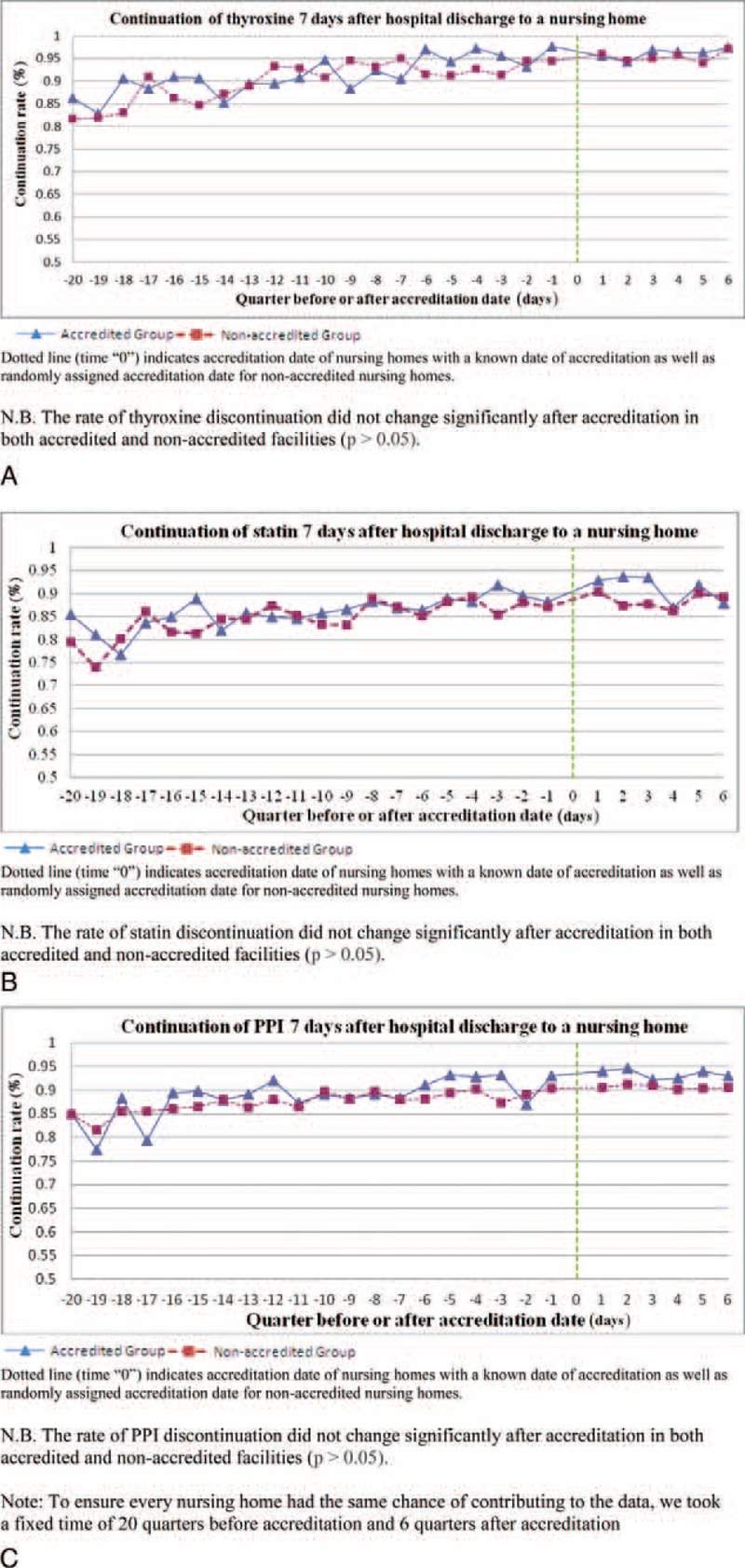
(A) Time series analysis of continuation of thyroxine 7 days after hospital discharge to a nursing home (fiscal years 2003–2011). (B) Time series analysis of statin continuation 7 days after hospital discharge to a nursing home (fiscal years 2003–2011). (C) Time series analysis of PPI continuation 7 days after hospital discharge to a nursing home (fiscal year 2003–2011). PPI = proton pump inhibitor.

The time series analysis showed no statistically significant difference (all *P* values were >0.05) in the observed rates of medication discontinuation for both accredited and nonaccredited facilities. As such, nursing home accreditation did not have a significant impact on medication discontinuation rates in nursing home residents after hospital discharge for any of the 3 drug groups (Figure 3A–C).

### Comment

Our study analyzed the effect of a national medication reconciliation accreditation requirement for nursing homes on rates of unintentional medication discontinuation after hospital discharge. We evaluated this effect from 2003 to 2012 in a population-based study of >100,000 hospitalized older adults who were admitted from and discharged to a nursing home in Ontario, Canada. We studied 3 evidence-based medications used for the treatment of chronic disease, which were identified as consensus quality indicators for medication continuity between hospitals and nursing homes: thyroxine, statins, and PPIs.^28–31^ We found that overall rates of unintentional medication discontinuation improved markedly over the 9-year study period. However, a time series analysis revealed that the 2008 introduction of medication reconciliation as a required practice for nursing home accreditation in Canada had no statistically significant impact on rates of unintentional medication discontinuation, suggesting the improvements may be explained by global improvements over time.

Our findings are important because much of the research on transition of care-related medication discontinuation has centered on the transition between acute care and community settings. An earlier and much smaller study of 71 bidirectional transfers between 4 nursing homes and 2 academic hospitals reported that ADEs attributable to medication changes occurred during 14 (20%) transfers, and 7 (50%) of these events were caused by discontinuations in drug use, representing a 3.5% risk of ADE per discontinuation in drug use.^11^ Here we analyzed unintentional medication discontinuation in the nursing home population on a system-wide basis over a 9-year period with a focus on important long-term medications for chronic diseases. We found that rates of unintentional medication discontinuation more than halved over the study period, suggesting fewer residents are now at risk for this potential ADE.

Besides reporting on an underresearched area of patient safety, our results signify important improvements in medication discontinuity between acute care hospitals and nursing homes from 2003 to 2012. Although the 2008 introduction of a national medication reconciliation accreditation requirement did not appear to have an impact on rates of medication discontinuation, we suspect that the overall improvement observed was reflective of multiple processes and not just 1 individual intervention. Indeed, the issue of patient and medication safety gained widespread attention with the 2004 launch of the 100,000 Lives Campaign—a key intervention being the prevention of ADEs through medication reconciliation.^41^ In Canada, the Canadian Patient Safety Institute launched the Safer Healthcare Now! medication reconciliation program in 2005. Concurrently, robust research emerged reporting on the positive effects of medication reconciliation programs especially during hospital discharge.^42,43^ Additionally, health and social care professional schools were focusing on patient safety, with most medical schools and residency programs offering patient safety training.^44^ Taken together, these developments may have led to a broader change of mindset, resulting in global improvements in patient and medication safety that complemented formalized quality improvement initiatives. As well, it is possible that our study was not able to identify an effect of the new accreditation standard precisely because unintentional medical discontinuation was less frequent by the time medication reconciliation became a required practice. Moreover, implementation of the accreditation standard may have lagged behind typical hospital practice. This could occur if nursing homes and hospitals were anticipating the introduction of an accreditation requirement, and medication reconciliation was already widely adopted so that the new standard alone did not result in any large improvement. Finally, unintentional discontinuation of medications is not the only metric for assessing the effectiveness of medical reconciliation programs.

The relationship between policy and health care system change is complex. Elsewhere, discrete and system-wide legislation has not necessarily resulted in the desired outcomes expected by policymakers. This includes regulatory action on benzodiazepine prescribing,^45^ the public release of data on cardiac quality indicators,^46^ and surgery safety checklists.^47^ Unlike a controlled study, policy occurs in a real-world setting, in which multiple interacting processes exist that can affect the fidelity of health care systems to an intervention. This may explain the nonlinear relationship between medical reconciliation accreditation requirements and rates of unintentional medication discontinuation seen in our study.

We must acknowledge several important limitations in our study design and analysis. Our population-based cohort study used linked administrative records that can evaluate associations but cannot prove causality. However, the data sources used have previously demonstrated good reliability, and the methodology applied has been used in previous work studying medications in elderly residents of nursing homes.^23,24^ Our study design also precluded us from determining whether medication discontinuation was truly unintentional. Indeed, many in our study population met the definition for polypharmacy,^48^ with participants taking a mean of nearly 16 different medications in the year prior to their index hospitalization. We acknowledge, that some medications may have been intentionally and appropriately discontinued by health care practitioners performing medication reviews in order to manage polypharmacy and medication side effects.^49^ Although statins and PPIs might be appropriate choices for intentional discontinuation, it would be unusual for thyroxine to be discontinued if a patient was on it continuously prior to hospitalization. However, the discontinuation trends observed for thyroxine mirrored those for statins and PPIs over the time period of the study. As well, we mitigated the issue of intentional medication discontinuation by including medications used for the management of chronic disease and by requiring ≥1 year of continuous use to exclude the possibility that the drug was discontinued due to a completed course of treatment. By only including patients with a long duration of continuous use, we also attempted to minimize confounding from patient nonadherence, which typically occurs within the first 6 months of treatment.^25,50^ Finally, in order to minimize discontinuation secondary to medication side effects, we excluded patients with medication-specific complications.

Another important drawback is that we excluded nursing home residents who died within 7 days after hospital discharge. Because unintentional medication discontinuation may be associated with adverse health outcomes and mortality,^3^ our exclusion of these subjects may have caused an underestimation of the true rates of medication discontinuation. We should also consider an alternative possibility, in which medications were intentionally discontinued among patients whose goals of care changed to a palliative approach.

Additionally, our analysis of the impact of the medication reconciliation accreditation requirement on unintentional medication discontinuation was limited by several factors. First, although medication reconciliation for nursing homes was not introduced as a required practice by Accreditation Canada until 2008, medication reconciliation had already been well established elsewhere.^42,43^ It is therefore possible that some nursing homes were pursuing medication reconciliation prior to their accreditation, therefore biasing toward a null effect of accreditation in our time series analysis. Furthermore, medication reconciliation was but one of several required practices for accreditation, and nursing homes could be accredited without meeting this standard. Indeed, compliance rates with the Accreditation Canada medication reconciliation required practice in Ontario increased from 55% in 2009 to 95% in 2012 at admission to nursing home (Accreditation Canada, personal communication, January 15, 2014). Moreover, the accreditation of nursing home providers with multiple sites may not reflect the practices at all sites.

As in any noncontrolled evaluation, we cannot exclude the possibility of confounding cointerventions. However, we are not aware of other relevant competing policy changes in Ontario, Canada, during our study period. Furthermore, we focused only on accreditation during the initial 3-year time frame after the introduction of the policy to allow a reasonable time to implement medication reconciliation programs. We cannot exclude the possibility that some nursing homes classified as nonaccredited were later accredited after this period. However, we did not observe any large increases in the numbers of newly accredited facilities after that date. As well, effect size may have been underestimated as a result of lag time between policy change, institutional uptake, and systems change. Finally, a time series analysis is known to be a conservative statistical technique to assess impacts of an intervention over time.^34,35^ Indeed, ceiling effects as a result of already high medication continuation rates before the accreditation intervention may have also played a role.

Despite these limitations, we feel confident in the validity of our principal finding that from 2003 to 2012, there were dramatic improvements in rates of unintentional medication discontinuation among hospitalized older adults who were admitted from and discharged to nursing homes. Although the medication reconciliation accreditation requirement that emerged in 2008 did not appear to have an impact on rates of medication discontinuation, we suspect that the global progress observed was reflective of multiple processes and not 1 individual intervention. In no way can we conclude that the accreditation requirement for medication reconciliation had no effect on the observed improvement.

Overall, the nearly decade-long gains in medication continuity are impressive and should motivate continued focus on patient and medication safety, especially in nursing homes. Future research efforts could focus on patient and facility characteristics that may influence transition of care-related medication discontinuation. Finally, as leaders in patient safety and policy grapple with other pressing patient safety issues, our findings highlight that policy change alone may not drive a large system change by itself. However, these public policy changes may serve as important catalysts for other drivers of system-wide improvement processes. To this point, we see the adoption of medication reconciliation into accreditation requirements as a major step forward toward improving the safety of patients across transitions of care.
