# Unintentional Discontinuation of Chronic Medications for Seniors in Nursing Homes: Evaluation of a National Medication Reconciliation Accreditation Requirement Using a Population-Based Cohort Study: Erratum

**DOI:** 10.1097/01.md.0000470335.28349.f2

**Published:** 2015-08-07

In the article “Unintentional Discontinuation of Chronic Medications for Seniors in Nursing Homes: Evaluation of a National Medication Reconciliation Accreditation Requirement Using a Population-Based Cohort Study”,^1^ which appeared in Volume 94, Issue 25 of *Medicine*, Figure 2 was presented incorrectly. The article has since been corrected online.
